# Tetra­imidazolium piperazinediium bis­(benzene-1,3,5-tricarboxyl­ate) dihydrate

**DOI:** 10.1107/S1600536810041310

**Published:** 2010-10-23

**Authors:** Ping-xian Liu, Zhi-an Li, Dao-yong Chen

**Affiliations:** a The State Key Laboratory Breeding Base of Basic Science of Stomatology, Hubei Province and School of Stomatology, Wuhan University 430072, People’s Republic of China; b College of Chemistry and Molecular Science, Wuhan University 430072, People’s Republic of China

## Abstract

During the crystallization of the title compound, 4C_3_H_5_N_2_^+^·C_4_H_12_N_2_^+^·2C_9_H_3_O_6_^3−^·2H_2_O, the acidic protons were transferred to the imidazole and piperazine N atoms, forming the final 4:1:2:2 hydrated mixed salt. The mean planes of the three carboxyl­ate groups in the anion are twisted with respect to the the central benzene ring, making dihedral angles of 13.5 (1), 14.5 (1) and 16.9 (1)°. In the crystal, the component ions are linked into a three-dimensional network by a combination of inter­molecular N—H⋯O, O—H⋯O and weak C—H⋯O hydrogen bonds. Further stabilization is provided by π-π stacking inter­actions with centroid–centroid distances of 3.393 (2) Å and weak C=O⋯π inter­actions [O–centroid = 3.363 (2) Å].

## Related literature

For applications of multi-component piperazine compounds, see: Jacobs *et al.* (2009 ▶); Oswald *et al.* (2002 ▶); Wang & Jia (2008 ▶). For examples of compounds containing weak anion–π inter­actions, see: Schottel *et al.* (2008 ▶); Gao *et al.* (2009 ▶).
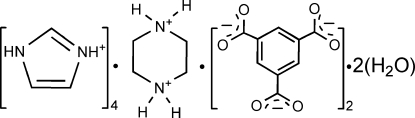


## Experimental

### 

#### Crystal data

4C_3_H_5_N_2_^+^·C_4_H_12_N_2_^+^·2C_9_H_3_O_6_^3−^·2H_2_O
*M* *_r_* = 814.78
Triclinic, 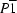

*a* = 7.1548 (4) Å
*b* = 9.9424 (5) Å
*c* = 13.3567 (7) Å
α = 96.895 (1)°
β = 95.201 (1)°
γ = 101.439 (2)°
*V* = 918.11 (8) Å^3^
*Z* = 1
Mo *K*α radiation
μ = 0.12 mm^−1^
*T* = 298 K
0.20 × 0.13 × 0.10 mm

#### Data collection

Bruker SMART APEX CCD area-detector diffractometer
Absorption correction: multi-scan (*SADABS*; Sheldrick, 1996 ▶) *T*_min_ = 0.967, *T*_max_ = 0.989
10341 measured reflections
3925 independent reflections
2757 reflections with *I* > 2σ(*I*)
*R*_int_ = 0.029

#### Refinement

*R*[*F* ^2^ > 2σ(*F* ^2^)] = 0.045
*wR*(*F* ^2^) = 0.122
*S* = 1.04
3925 reflections
286 parameters
H atoms treated by a mixture of independent and constrained refinement
Δρ_max_ = 0.31 e Å^−3^
Δρ_min_ = −0.19 e Å^−3^

### 

Data collection: *SMART* (Bruker, 2001 ▶); cell refinement: *SAINT-Plus* (Bruker, 2001 ▶); data reduction: *SAINT-Plus*; program(s) used to solve structure: *SHELXS97* (Sheldrick, 2008 ▶); program(s) used to refine structure: *SHELXL97* (Sheldrick, 2008 ▶); molecular graphics: *SHELXTL* (Sheldrick, 2008 ▶) and *PLATON* (Spek, 2009 ▶); software used to prepare material for publication: *PLATON*.

## Supplementary Material

Crystal structure: contains datablocks global, I. DOI: 10.1107/S1600536810041310/lh5148sup1.cif

Structure factors: contains datablocks I. DOI: 10.1107/S1600536810041310/lh5148Isup2.hkl

Additional supplementary materials:  crystallographic information; 3D view; checkCIF report

## Figures and Tables

**Table 1:** Hydrogen-bond geometry (Å, °)

*D*—H⋯*A*	*D*—H	H⋯*A*	*D*⋯*A*	*D*—H⋯*A*
N1—H1*A*⋯O2	0.87 (2)	1.83 (2)	2.6870 (18)	166.9 (19)
N2—H2*A*⋯O5^i^	0.90 (2)	1.86 (2)	2.7522 (19)	177.4 (19)
N3—H3*A*⋯O6	0.915 (19)	1.80 (2)	2.701 (2)	168.8 (18)
N4—H4*A*⋯O2^ii^	0.93 (2)	1.74 (2)	2.668 (2)	176.6 (18)
N5—H5*A*⋯O3^iii^	0.98 (2)	1.83 (2)	2.7872 (19)	166.7 (17)
N5—H5*A*⋯O4^iii^	0.98 (2)	2.60 (2)	3.366 (2)	135.7 (14)
N5—H5*B*⋯O5	0.91 (2)	2.19 (2)	2.985 (2)	145.2 (16)
N5—H5*B*⋯O6	0.91 (2)	2.10 (2)	2.888 (2)	145.1 (18)
O7—H7*A*⋯O1	0.86 (3)	1.86 (3)	2.7184 (17)	171 (2)
O7—H7*B*⋯O3^iv^	0.90 (3)	1.95 (3)	2.840 (2)	169 (2)
C10—H10⋯O7	0.93	2.35	3.217 (3)	156
C12—H12⋯O4^i^	0.93	2.32	3.244 (2)	175
C13—H13⋯O1	0.93	2.36	3.267 (2)	164
C14—H14⋯O3^iv^	0.93	2.51	3.372 (2)	154
C15—H15⋯O7^v^	0.93	2.38	3.186 (2)	145
C16—H16*B*⋯O4^vi^	0.97	2.32	3.200 (2)	150
C16—H16*A*⋯O7^v^	0.97	2.48	3.207 (2)	132

Symmetry codes: (i) 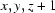; (ii) 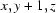; (iii) 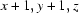; (iv) 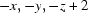; (v) 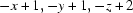; (vi) 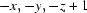.

**Fig. 1.**
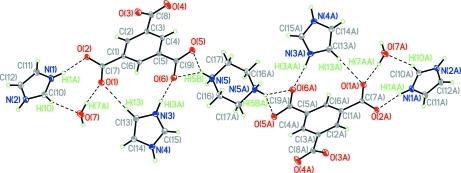
The formula unit of the title compound. The displacement ellipsoids are drawn at the 30% probability level. Hydrogen bonds are shown as dashed lines. Atoms marked with 'A' are at the position of (1 - x, 1 - y, 1 - z).

**Fig. 2.**
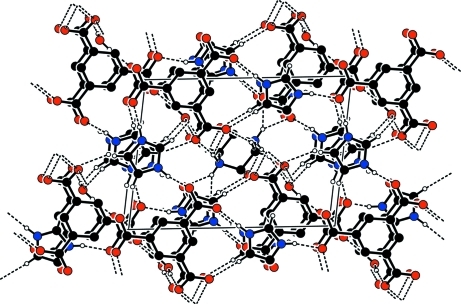
Part of the title crystal structure, showing the three-dimensional network linked by N—H···O, O—H···O and C—H···O hydrogen bonds represented by dashed lines. For the sake of clarity, the H atoms not involved in the hydrogen-bonds pattern have been omitted. Color code: C, black; H, white; N, blue; O, red.

## supplementary crystallographic information

### Comment

Piperazine has been used in the manufacture of anthelmintic vermicide. In order to improve its pharmaceutical effect, piperazine has been made into tablets containing two or more components (Jacobs *et al.*, 2009; Oswald *et al.*, 2002; Wang & Jia, 2008). In this paper, we report a new four-component piperazine containing adduct and the crystal structure is presented herein.

The formula unit of the title compound is shown in Fig. 1. The asymmetric unit is composed of two imidazolium cations, half a piperazinium cation, one benzene 1,3,5-tricarboxylate trianion and one water molecule. During the crystallization, the carboxylic acid protons of benzene-1,3,5-tricarboxylic acid were transferred to the imidazole and piperazine nitrogen atoms, forming the 4:1:2:2 organic adduct (imidazolium: piperazinium: benzene 1,3,5-tricarboxylate: water). Delocalization of charges on the the imidazolium and the benzene-1,3,5-tricarboxylate ions are reflected in the bond distances of C15—N3, C15—N4, C9—O5 and C9—O6. The mean planes of the three carboxylate groups in the anion twisted away from the central benzene ring with dihedral angles of 13.5 (1)°, 14.5 (1)° and 16.9 (1)°.

In the crystal packing, the component ions are linked into a complex three-dimensional network by a combination of N—H···O, O—H···O and C—H···O hydrogen bonds (Table 1 and Figure 2). A *PLATON* (Spek, 2009) analysis shows that the crystal structure is  further consolidated by a π···π interaction [*Cg*1···*Cg*1(1 - *x*, 1 - *y*, 2 - *z*) = 3.393 (2) Å, dihedral angle = 0°, *Cg*1 is the centroid defined by atoms N3/N4/C13—C15] and a C=O···π interaction (C9···*Cg*2 = 3.472 (2) Å, O5···*Cg*2 = 3.363 (2)Å and C9=O5···*Cg*2 = 84.4 (2)°, *Cg*2 is th centroid defined by atoms N1/N2/C10—C12). These types of weak interactions have previously been studied (Gao *et al.*, 2009, Schottel *et al.*, 2008).

### Experimental

All the reagents and solvents were used as obtained without further purification. Piperazine hexahydrate (0.2 mmol, 38.8 mg), imidazole (0.2 mmol, 13.6 mg) and benzene 1,3,5-tricarboxylic acid (0.1 mmol, 42.0 mg) were dissolved in 95% methanol (20 ml). The resulting colorless solution was kept in air for two weeks. Colorless blocks of the title compound suitable for single-crystal X-ray diffraction analysis were grown by slow evaporation of the solution at the bottom of the vessel.

### Refinement

H atoms bonded to C atoms were positioned geometrically with C–H = 0.93Å (aromatic) and 0.97 Å (methylene) and refined in a riding-model approximation [*U*_iso_(H) = 1.2*U*_eq_(C). H atoms bonded to N and O atoms were found in Fourier difference maps with N—H and were refined freely  with *U*_iso_(H)= 1.2*U*_eq_(N) or 1.5*U*_eq_(O).

### Crystal data

**Table:**

4C_3_H_5_N_2_^+^·C_4_H_12_N_2_^+^·2C_9_H_3_O_6_^3^^−^·2H_2_O	*Z* = 1
*M**_r_* = 814.78	*F*(000) = 428
Triclinic, *P*1	*D*_x_ = 1.474 Mg m^−^^3^
Hall symbol: -P 1	Mo *K*α radiation, λ = 0.71073 Å
*a* = 7.1548 (4) Å	Cell parameters from 2102 reflections
*b* = 9.9424 (5) Å	θ = 2.4–23.2°
*c* = 13.3567 (7) Å	µ = 0.12 mm^−^^1^
α = 96.895 (1)°	*T* = 298 K
β = 95.201 (1)°	Block, colorless
γ = 101.439 (2)°	0.20 × 0.13 × 0.10 mm
*V* = 918.11 (8) Å^3^	

### Data collection

**Table:**

Bruker SMART APEX CCD area-detector diffractometer	3925 independent reflections
Radiation source: fine focus sealed Siemens Mo tube	2757 reflections with *I* > 2σ(*I*)
graphite	*R*_int_ = 0.029
0.3° wide ω scans	θ_max_ = 27.0°, θ_min_ = 2.4°
Absorption correction: multi-scan (*SADABS*; Sheldrick, 1996)	*h* = −8→9
*T*_min_ = 0.967, *T*_max_ = 0.989	*k* = −11→12
10341 measured reflections	*l* = −17→17

### Refinement

**Table:**

Refinement on *F*^2^	Primary atom site location: structure-invariant direct methods
Least-squares matrix: full	Secondary atom site location: difference Fourier map
*R*[*F*^2^ > 2σ(*F*^2^)] = 0.045	Hydrogen site location: inferred from neighbouring sites
*wR*(*F*^2^) = 0.122	H atoms treated by a mixture of independent and constrained refinement
*S* = 1.04	*w* = 1/[σ^2^(*F*_o_^2^) + (0.0631*P*)^2^ + 0.0369*P*] where *P* = (*F*_o_^2^ + 2*F*_c_^2^)/3
3925 reflections	(Δ/σ)_max_ = 0.001
286 parameters	Δρ_max_ = 0.31 e Å^−^^3^
0 restraints	Δρ_min_ = −0.18 e Å^−^^3^

### Special details

**Table:**

Geometry. All e.s.d.'s (except the e.s.d. in the dihedral angle between two l.s. planes) are estimated using the full covariance matrix. The cell e.s.d.'s are taken into account individually in the estimation of e.s.d.'s in distances, angles and torsion angles; correlations between e.s.d.'s in cell parameters are only used when they are defined by crystal symmetry. An approximate (isotropic) treatment of cell e.s.d.'s is used for estimating e.s.d.'s involving l.s. planes.
Refinement. Refinement of *F*^2^ against ALL reflections. The weighted *R*-factor *wR* and goodness of fit *S* are based on *F*^2^, conventional *R*-factors *R* are based on *F*, with *F* set to zero for negative *F*^2^. The threshold expression of *F*^2^ > σ(*F*^2^) is used only for calculating *R*-factors(gt) *etc*. and is not relevant to the choice of reflections for refinement. *R*-factors based on *F*^2^ are statistically about twice as large as those based on *F*, and *R*- factors based on ALL data will be even larger.

### Fractional atomic coordinates and isotropic or equivalent isotropic displacement parameters (Å^2^)

**Table:**

	*x*	*y*	*z*	*U*_iso_*/*U*_eq_	
C1	0.2128 (2)	−0.05764 (17)	0.89657 (11)	0.0263 (4)	
C2	0.1004 (2)	−0.17749 (17)	0.84080 (11)	0.0263 (4)	
H2	0.0409	−0.2481	0.8743	0.032*	
C3	0.0756 (2)	−0.19338 (17)	0.73487 (11)	0.0274 (4)	
C4	0.1609 (2)	−0.08588 (18)	0.68651 (12)	0.0295 (4)	
H4	0.1465	−0.0964	0.6159	0.035*	
C5	0.2676 (2)	0.03761 (17)	0.74101 (12)	0.0267 (4)	
C6	0.2947 (2)	0.04896 (17)	0.84640 (12)	0.0274 (4)	
H6	0.3694	0.1297	0.8838	0.033*	
C7	0.2458 (2)	−0.04026 (18)	1.01134 (12)	0.0297 (4)	
C8	−0.0459 (2)	−0.32408 (19)	0.67241 (12)	0.0332 (4)	
C9	0.3502 (2)	0.15651 (19)	0.68720 (13)	0.0310 (4)	
C10	0.2728 (3)	−0.0067 (2)	1.32738 (13)	0.0391 (5)	
H10	0.3430	0.0790	1.3175	0.047*	
C11	0.1114 (3)	−0.2190 (2)	1.29964 (14)	0.0432 (5)	
H11	0.0505	−0.3063	1.2661	0.052*	
C12	0.1222 (3)	−0.1753 (2)	1.39965 (13)	0.0421 (5)	
H12	0.0702	−0.2261	1.4484	0.051*	
C13	0.2479 (3)	0.3752 (2)	0.99158 (14)	0.0401 (5)	
H13	0.2379	0.2876	1.0111	0.048*	
C14	0.2247 (3)	0.4896 (2)	1.04845 (14)	0.0396 (5)	
H14	0.1949	0.4961	1.1150	0.048*	
C15	0.2920 (3)	0.5449 (2)	0.90127 (14)	0.0400 (5)	
H15	0.3176	0.5954	0.8480	0.048*	
C16	0.3894 (3)	0.4987 (2)	0.58396 (13)	0.0422 (5)	
H16A	0.3773	0.5318	0.6539	0.051*	
H16B	0.2710	0.4341	0.5557	0.051*	
C17	0.5803 (3)	0.3814 (2)	0.47501 (14)	0.0462 (5)	
H17A	0.4688	0.3126	0.4428	0.055*	
H17B	0.6910	0.3387	0.4747	0.055*	
N1	0.2057 (2)	−0.11215 (17)	1.25638 (11)	0.0396 (4)	
H1A	0.217 (3)	−0.111 (2)	1.1923 (16)	0.048*	
N2	0.2245 (2)	−0.04171 (17)	1.41557 (11)	0.0389 (4)	
H2A	0.265 (3)	0.014 (2)	1.4745 (16)	0.047*	
N3	0.2891 (2)	0.41179 (16)	0.89917 (12)	0.0374 (4)	
H3A	0.320 (3)	0.355 (2)	0.8468 (14)	0.045*	
N4	0.2528 (2)	0.59528 (17)	0.99092 (11)	0.0361 (4)	
H4A	0.240 (3)	0.686 (2)	1.0107 (14)	0.043*	
N5	0.5516 (2)	0.42669 (17)	0.58090 (11)	0.0388 (4)	
H5A	0.667 (3)	0.486 (2)	0.6204 (14)	0.047*	
H5B	0.520 (3)	0.350 (2)	0.6120 (15)	0.047*	
O1	0.3047 (2)	0.07841 (13)	1.05718 (9)	0.0488 (4)	
O2	0.21058 (18)	−0.14867 (12)	1.05409 (8)	0.0379 (3)	
O3	−0.15144 (18)	−0.40803 (13)	0.71833 (9)	0.0450 (4)	
O4	−0.0363 (2)	−0.34118 (16)	0.58017 (9)	0.0617 (5)	
O5	0.3573 (2)	0.13542 (13)	0.59351 (9)	0.0436 (3)	
O6	0.41142 (19)	0.27435 (12)	0.73950 (9)	0.0415 (3)	
O7	0.4407 (2)	0.26393 (16)	1.22724 (10)	0.0498 (4)	
H7A	0.407 (3)	0.201 (3)	1.1749 (19)	0.075*	
H7B	0.342 (3)	0.306 (3)	1.2364 (18)	0.075*	

### Atomic displacement parameters (Å^2^)

**Table:**

	*U*^11^	*U*^22^	*U*^33^	*U*^12^	*U*^13^	*U*^23^
C1	0.0326 (9)	0.0259 (9)	0.0207 (8)	0.0069 (7)	0.0028 (6)	0.0045 (6)
C2	0.0329 (9)	0.0236 (9)	0.0223 (8)	0.0025 (7)	0.0046 (6)	0.0073 (6)
C3	0.0293 (8)	0.0277 (10)	0.0243 (8)	0.0042 (7)	0.0023 (6)	0.0038 (7)
C4	0.0368 (9)	0.0330 (10)	0.0186 (7)	0.0064 (8)	0.0040 (6)	0.0047 (7)
C5	0.0301 (9)	0.0256 (9)	0.0265 (8)	0.0071 (7)	0.0068 (6)	0.0069 (7)
C6	0.0330 (9)	0.0218 (9)	0.0261 (8)	0.0036 (7)	0.0025 (7)	0.0027 (7)
C7	0.0397 (10)	0.0282 (10)	0.0213 (8)	0.0080 (8)	0.0034 (7)	0.0026 (7)
C8	0.0360 (9)	0.0336 (11)	0.0256 (8)	0.0009 (8)	−0.0006 (7)	0.0013 (7)
C9	0.0357 (9)	0.0301 (10)	0.0307 (9)	0.0095 (8)	0.0088 (7)	0.0103 (7)
C10	0.0498 (11)	0.0398 (12)	0.0308 (9)	0.0124 (9)	0.0099 (8)	0.0090 (8)
C11	0.0501 (11)	0.0425 (12)	0.0332 (10)	0.0025 (9)	0.0019 (8)	0.0040 (9)
C12	0.0477 (11)	0.0478 (13)	0.0322 (10)	0.0064 (10)	0.0094 (8)	0.0140 (9)
C13	0.0473 (11)	0.0327 (11)	0.0427 (10)	0.0080 (9)	0.0089 (8)	0.0128 (9)
C14	0.0465 (11)	0.0394 (12)	0.0352 (10)	0.0105 (9)	0.0087 (8)	0.0086 (8)
C15	0.0500 (11)	0.0342 (12)	0.0376 (10)	0.0081 (9)	0.0112 (8)	0.0097 (8)
C16	0.0441 (11)	0.0506 (13)	0.0269 (9)	−0.0014 (9)	0.0040 (8)	0.0040 (8)
C17	0.0566 (12)	0.0361 (12)	0.0432 (11)	0.0085 (10)	−0.0001 (9)	0.0013 (9)
N1	0.0508 (10)	0.0500 (11)	0.0216 (7)	0.0155 (8)	0.0074 (7)	0.0090 (7)
N2	0.0487 (9)	0.0454 (11)	0.0233 (7)	0.0121 (8)	0.0055 (7)	0.0035 (7)
N3	0.0446 (9)	0.0289 (9)	0.0390 (9)	0.0081 (7)	0.0091 (7)	0.0020 (7)
N4	0.0434 (9)	0.0264 (9)	0.0391 (8)	0.0086 (7)	0.0076 (7)	0.0037 (7)
N5	0.0455 (10)	0.0309 (9)	0.0344 (8)	−0.0067 (8)	−0.0049 (7)	0.0143 (7)
O1	0.0874 (11)	0.0259 (7)	0.0268 (6)	0.0060 (7)	−0.0025 (6)	−0.0047 (5)
O2	0.0642 (9)	0.0285 (7)	0.0213 (6)	0.0072 (6)	0.0072 (5)	0.0076 (5)
O3	0.0521 (8)	0.0360 (8)	0.0371 (7)	−0.0124 (6)	0.0057 (6)	0.0016 (6)
O4	0.0783 (11)	0.0614 (10)	0.0263 (7)	−0.0223 (8)	0.0034 (7)	−0.0059 (6)
O5	0.0648 (9)	0.0375 (8)	0.0297 (7)	0.0058 (6)	0.0163 (6)	0.0107 (5)
O6	0.0592 (8)	0.0252 (7)	0.0393 (7)	0.0009 (6)	0.0146 (6)	0.0079 (6)
O7	0.0637 (10)	0.0433 (9)	0.0349 (7)	−0.0004 (7)	0.0066 (7)	−0.0058 (6)

### Geometric parameters (Å, °)

**Table:**

C1—C6	1.382 (2)	C12—H12	0.9300
C1—C2	1.385 (2)	C13—C14	1.337 (3)
C1—C7	1.513 (2)	C13—N3	1.369 (2)
C2—C3	1.396 (2)	C13—H13	0.9300
C2—H2	0.9300	C14—N4	1.369 (2)
C3—C4	1.382 (2)	C14—H14	0.9300
C3—C8	1.517 (2)	C15—N3	1.317 (2)
C4—C5	1.390 (2)	C15—N4	1.318 (2)
C4—H4	0.9300	C15—H15	0.9300
C5—C6	1.391 (2)	C16—N5	1.481 (3)
C5—C9	1.506 (2)	C16—C17^i^	1.497 (3)
C6—H6	0.9300	C16—H16A	0.9700
C7—O1	1.236 (2)	C16—H16B	0.9700
C7—O2	1.272 (2)	C17—N5	1.477 (2)
C8—O4	1.233 (2)	C17—C16^i^	1.497 (3)
C8—O3	1.265 (2)	C17—H17A	0.9700
C9—O5	1.251 (2)	C17—H17B	0.9700
C9—O6	1.264 (2)	N1—H1A	0.87 (2)
C10—N1	1.307 (2)	N2—H2A	0.90 (2)
C10—N2	1.324 (2)	N3—H3A	0.915 (19)
C10—H10	0.9300	N4—H4A	0.93 (2)
C11—C12	1.345 (3)	N5—H5A	0.98 (2)
C11—N1	1.361 (2)	N5—H5B	0.91 (2)
C11—H11	0.9300	O7—H7A	0.86 (3)
C12—N2	1.365 (2)	O7—H7B	0.90 (3)
			
C6—C1—C2	119.32 (14)	N3—C13—H13	126.6
C6—C1—C7	119.29 (14)	C13—C14—N4	107.51 (16)
C2—C1—C7	121.39 (15)	C13—C14—H14	126.2
C1—C2—C3	120.66 (15)	N4—C14—H14	126.2
C1—C2—H2	119.7	N3—C15—N4	109.13 (17)
C3—C2—H2	119.7	N3—C15—H15	125.4
C4—C3—C2	118.85 (14)	N4—C15—H15	125.4
C4—C3—C8	119.66 (14)	N5—C16—C17^i^	110.90 (16)
C2—C3—C8	121.48 (15)	N5—C16—H16A	109.5
C3—C4—C5	121.49 (14)	C17^i^—C16—H16A	109.5
C3—C4—H4	119.3	N5—C16—H16B	109.5
C5—C4—H4	119.3	C17^i^—C16—H16B	109.5
C4—C5—C6	118.38 (15)	H16A—C16—H16B	108.0
C4—C5—C9	120.74 (14)	N5—C17—C16^i^	111.07 (16)
C6—C5—C9	120.88 (15)	N5—C17—H17A	109.4
C1—C6—C5	121.22 (15)	C16^i^—C17—H17A	109.4
C1—C6—H6	119.4	N5—C17—H17B	109.4
C5—C6—H6	119.4	C16^i^—C17—H17B	109.4
O1—C7—O2	124.44 (15)	H17A—C17—H17B	108.0
O1—C7—C1	117.75 (16)	C10—N1—C11	108.59 (15)
O2—C7—C1	117.81 (14)	C10—N1—H1A	124.3 (14)
O4—C8—O3	124.28 (16)	C11—N1—H1A	127.1 (14)
O4—C8—C3	117.93 (16)	C10—N2—C12	108.49 (16)
O3—C8—C3	117.79 (14)	C10—N2—H2A	122.6 (13)
O5—C9—O6	122.58 (16)	C12—N2—H2A	128.7 (13)
O5—C9—C5	119.37 (16)	C15—N3—C13	108.45 (16)
O6—C9—C5	118.05 (14)	C15—N3—H3A	125.6 (13)
N1—C10—N2	108.95 (18)	C13—N3—H3A	125.7 (12)
N1—C10—H10	125.5	C15—N4—C14	108.03 (16)
N2—C10—H10	125.5	C15—N4—H4A	126.1 (12)
C12—C11—N1	107.52 (17)	C14—N4—H4A	125.8 (12)
C12—C11—H11	126.2	C17—N5—C16	110.82 (14)
N1—C11—H11	126.2	C17—N5—H5A	113.0 (11)
C11—C12—N2	106.45 (17)	C16—N5—H5A	110.0 (11)
C11—C12—H12	126.8	C17—N5—H5B	108.4 (13)
N2—C12—H12	126.8	C16—N5—H5B	107.1 (13)
C14—C13—N3	106.88 (17)	H5A—N5—H5B	107.3 (16)
C14—C13—H13	126.6	H7A—O7—H7B	108 (2)
			
C6—C1—C2—C3	2.5 (2)	C4—C3—C8—O3	−166.35 (16)
C7—C1—C2—C3	−178.26 (15)	C2—C3—C8—O3	12.2 (3)
C1—C2—C3—C4	−1.7 (3)	C4—C5—C9—O5	−14.7 (3)
C1—C2—C3—C8	179.66 (15)	C6—C5—C9—O5	165.86 (16)
C2—C3—C4—C5	−1.0 (3)	C4—C5—C9—O6	165.85 (16)
C8—C3—C4—C5	177.65 (15)	C6—C5—C9—O6	−13.6 (2)
C3—C4—C5—C6	2.9 (3)	N1—C11—C12—N2	−0.3 (2)
C3—C4—C5—C9	−176.58 (15)	N3—C13—C14—N4	−0.3 (2)
C2—C1—C6—C5	−0.5 (2)	N2—C10—N1—C11	−0.1 (2)
C7—C1—C6—C5	−179.78 (15)	C12—C11—N1—C10	0.3 (2)
C4—C5—C6—C1	−2.1 (2)	N1—C10—N2—C12	0.0 (2)
C9—C5—C6—C1	177.32 (15)	C11—C12—N2—C10	0.2 (2)
C6—C1—C7—O1	17.1 (2)	N4—C15—N3—C13	−0.5 (2)
C2—C1—C7—O1	−162.20 (16)	C14—C13—N3—C15	0.5 (2)
C6—C1—C7—O2	−163.16 (15)	N3—C15—N4—C14	0.3 (2)
C2—C1—C7—O2	17.6 (2)	C13—C14—N4—C15	0.0 (2)
C4—C3—C8—O4	13.2 (3)	C16^i^—C17—N5—C16	56.2 (2)
C2—C3—C8—O4	−168.22 (17)	C17^i^—C16—N5—C17	−56.1 (2)

Symmetry codes: (i) −*x*+1, −*y*+1, −*z*+1.

### Hydrogen-bond geometry (Å, °)

**Table:**

*D*—H···*A*	*D*—H	H···*A*	*D*···*A*	*D*—H···*A*
N1—H1A···O2	0.87 (2)	1.83 (2)	2.6870 (18)	166.9 (19)
N2—H2A···O5^ii^	0.90 (2)	1.86 (2)	2.7522 (19)	177.4 (19)
N3—H3A···O6	0.915 (19)	1.80 (2)	2.701 (2)	168.8 (18)
N4—H4A···O2^iii^	0.93 (2)	1.74 (2)	2.668 (2)	176.6 (18)
N5—H5A···O3^iv^	0.98 (2)	1.83 (2)	2.7872 (19)	166.7 (17)
N5—H5A···O4^iv^	0.98 (2)	2.60 (2)	3.366 (2)	135.7 (14)
N5—H5B···O5	0.91 (2)	2.19 (2)	2.985 (2)	145.2 (16)
N5—H5B···O6	0.91 (2)	2.10 (2)	2.888 (2)	145.1 (18)
O7—H7A···O1	0.86 (3)	1.86 (3)	2.7184 (17)	171 (2)
O7—H7B···O3^v^	0.90 (3)	1.95 (3)	2.840 (2)	169 (2)
C10—H10···O7	0.93	2.35	3.217 (3)	156
C12—H12···O4^ii^	0.93	2.32	3.244 (2)	175
C13—H13···O1	0.93	2.36	3.267 (2)	164
C14—H14···O3^v^	0.93	2.51	3.372 (2)	154
C15—H15···O7^vi^	0.93	2.38	3.186 (2)	145
C16—H16B···O4^vii^	0.97	2.32	3.200 (2)	150
C16—H16A···O7^vi^	0.97	2.48	3.207 (2)	132

Symmetry codes: (ii) *x*, *y*, *z*+1; (iii) *x*, *y*+1, *z*; (iv) *x*+1, *y*+1, *z*; (v) −*x*, −*y*, −*z*+2; (vi) −*x*+1, −*y*+1, −*z*+2; (vii) −*x*, −*y*, −*z*+1.
